# Liver Regeneration and Aging: A Current Perspective

**DOI:** 10.1155/2011/526379

**Published:** 2011-09-08

**Authors:** Douglas L. Schmucker, Henry Sanchez

**Affiliations:** ^1^Department of Anatomy, School of Medicine, University of California, San Francisco, CA 94143, USA; ^2^Departments of Pathology, School of Medicine, University of California, San Francisco, CA 94143, USA

## Abstract

Many organ systems exhibit significant age-related deficits, but, based on studies in old rodents and elderly humans, the liver appears to be relatively protected from such changes. A remarkable feature of the liver is its capacity to regenerate its mass following partial hepatectomy. Reports suggests that aging compromises the liver's regenerative capacity, both in the rate and to the extent the organ's original volume is restored. There has been modest definitive information as to which cellular and molecular mechanisms regulating hepatic regeneration are affected by aging. Changes in hepatic sensitivity to growth factors, for example, epidermal growth factor (EGF), appear to influence regeneration in old animals. Studies have demonstrated (a) a 60% decline in EGF binding to hepatocyte plasma membranes, (b) reduced expression of the hepatic high affinity EGF receptor and (c) a block between G1 and S-phases of the cell cycle in old rats following EGF stimulation. Recent studies suggest that reduced phosphorylation and dimerization of the EGF receptor, critical steps in the activation of the extracellular signal-regulated kinase pathway and subsequent cell proliferation are responsible. Other studies have demonstrated that aging affects the upregulation of a Forkhead Box transcription factor, FoxM1B, which is essential for growth hormone-stimulated liver regeneration in hepatectomized mice. Aging appears to compromise liver regeneration by influencing several pathways, the result of which is a reduction in the rate of regeneration, but not in the capacity to restore the organ to its original volume.

## 1. Introduction

On the one hand, the liver, unlike most other organs, does not exhibit well-documented or marked changes in either structure or function during the aging process (see [1–4] for reviews). There have been few comprehensive studies on liver morphology during aging; most have been performed using rodent models and have been qualitative in nature. Studies using human liver tissue have suffered from dependence on postmortem samples or on samples from subjects diagnosed with liver disease. There is quantitative evidence that hepatocytes in males of one inbred rat strain (Fischer 344) increase in volume through maturity and, subsequently, become smaller such that the size of cells in immature and senescent animals is equivalent [5]. Other changes in hepatocellular structure include (a) a loss of smooth surfaced endoplasmic reticulum, (b) an increase in the volume of the dense body compartment, for example, secondary lysosomes, residual bodies, or lipofuscin, and (c) an increase in hepatocyte polyploidy (see [6] for a review). None of these age-related changes is manifested in significant declines in hepatic function(s). 

 On the other hand, there are data that demonstrate specific age-related changes, including a loss of hepatic volume and a decline in hepatic perfusion; both of which may affect certain liver functions, such as first pass pharmacokinetics [7, 8]. However, data from clinical liver function tests are inconclusive and fail to identify significant age-associated deficits in hepatic functions ([9, 10], see [2] for a review). Several studies, including our own, have demonstrated moderate age-related changes in biliary function, including decreased bile flow and bile acid secretion ([11], see [3] for a review).

The clearance of drugs that undergo mandatory Phase I hepatic metabolism may be compromised in the elderly (see [12] for a review). However, there is little evidence to support the hypothesis that reduced hepatic drug clearance reflects concomitant declines in the amounts or efficacies of human liver cytochrome P-450 isoforms [13]. A recent review argues that substantial age-related declines occur in hepatic Phase I drug metabolism as evidenced by reduced clearance of high-clearance drugs and that other data are misleading since most studies have assessed protein-bound plus free-drug concentrations (total clearance) rather than separating these two components [14]. It should be noted that drug clearance reflects several variables, including volume of distribution, protein binding, intrinsic hepatic metabolism, and renal clearance. While there are data suggesting that certain low-protein binding drugs exhibit reduced clearance in the elderly; the evidence for a similar decline in free-drug clearance is less viable. Studies by us and by others assessing the efficacy of specific P-450 isoforms across a broad age spectrum in humans suggest that intrinsic cytochrome P450-mediated hepatic drug metabolism, not total drug clearance, remains unchanged in humans as a function of age [12, 13]. The question of whether or not liver functions are compromised in senescent animals or elderly humans remains unclear. The late hepatologist, Hans Popper, stated that “aging exerts a limited effect on the constitutive functions of the liver and more on its response to extrahepatic factors…” [14].

**Figure 1:**
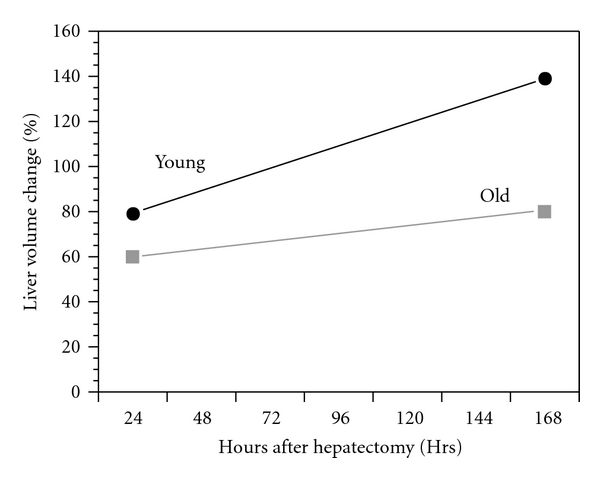
Effect of animal age on liver volume at two intervals following partial hepatectomy in young and old rats. Data derived from [15].

Perhaps a more clinically significant age-related change is a marked decline in the rate of hepatic regeneration following partial resection (hepatectomy) or chemically induced injury. This is manifested as a delay in hepatocyte proliferation following hepatectomy and is documented by a number of studies, including that of Popper [16] (Figure 1). The purpose of this brief review is to present a perspective of the current understanding of the cellular and molecular factors and mechanisms that contribute to the diminished hepatic regeneration rate in old-animal models and in elderly humans.

## 2. Basics of Liver Regeneration

**Figure 2:**
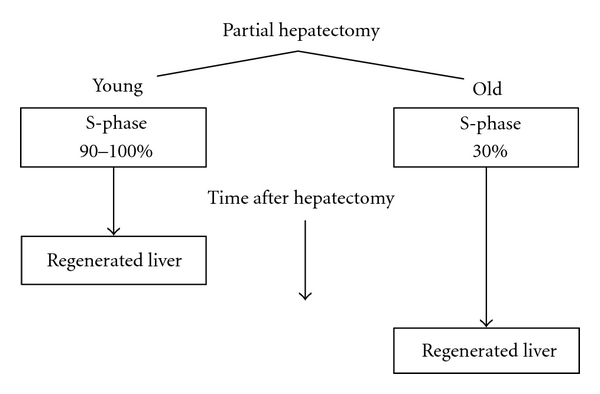
Effect of age on the number of hepatocytes entering S-phase and the rate of liver regeneration following partial hepatectomy.

First, hepatocytes constitute a population of highly differentiated, quiescent, yet intermitotic cells with few cells undergoing division at any one time, for example, approximately 1 mitotic figure per 20,000 cells in the resting liver. Second, the normal regenerative process reflects a global hyperplasia, that is, compensatory growth and division of existing hepatocytes, rather than a cellular hypertrophy or a primary stem cell response. Third, the regenerative process is highly regulated by signal transduction pathways (see [17] for a review). Fourth, the initiation of hepatocyte proliferation or liver regeneration requires the activation of specific cell cycle and mitogenic genes, as well as the repression of those genes responsible for inhibiting hepatocyte proliferation in the resting organ. The consensus is that fewer hepatocytes in senescent animals and elderly humans enter S-phase after partial hepatectomy in comparison to younger subjects, and those that do so less rapidly and that this age-related delay compromises the rate of liver regeneration (Figure 2).

## 3. Why the Concern about Compromised Liver Regeneration in the Elderly?

One reason is the marked increase in mortality due to liver disease in elderly subjects in comparison to younger populations. Regev and Schiff reported 3–5-fold increases in deaths due to liver diseases in the over 65 population versus those under 45 years of age [18]. Another basis for concern is the increased demand for donor livers for transplantation. This issue is complex since there is an effort to increase the age limits for liver donors and, to some extent, of liver recipients. On the one hand, there is evidence that livers from older donors may be less viable than those from young donors [19, 20]. Recipient age should also be a consideration since Fortner and Lincer reported that post-transplant mortality increases by 15% between 55 and 75 years of age [21]. On the other hand, there are data that suggest that the impact of age is modest with respect to recipient and graft survival, at least during the first few post-transplantation years. For example, both recipient and graft survival rates decline in elderly patients receiving livers from old donors by only 10–15% over the first three post-transplant years [22–24].

In summary, aging does impair liver regeneration with respect to the rate of hepatocyte proliferation following resection. The magnitude of this impairment does not seem to be excessive and, furthermore, may not be an impediment to the use of livers from elderly donors. However, before we can assess this option definitively, we need to clearly understand the cellular and molecular mechanisms that compromise liver regeneration in the elderly. There have been a number of hypotheses, some of which are discussed below. Perhaps the most comprehensive studies on this subject have been performed by Timchenko's group (see [17] for a review).

## 4. Age-Related Increases in Reactive Oxygen Species, Hepatocellular Residual Bodies, and Lipofuscin

One suggestion is that the documented age-related increase in residual bodies or lipofuscin in hepatocytes reflects an inability to eliminate cellular waste products and that this accumulation compromises normal cell activities (see [25] for a review). Our quantitative electron microscopic analysis demonstrated a 3-4-fold increase in the volume of this intracellular compartment during aging in rats [5]. However, since this compartment accounts for only about 1% of the total intracellular volume of hepatocytes, it seems inappropriate to assign this particular age-related shift a significant role in impeding hepatocyte proliferation and liver regeneration.

Reactive oxygen species (ROS) have been considered a causative factor responsible for a number of pathophysiological changes during aging. A recent study by Haga et al. implicates increased expression/phosphorylation of the adapter protein p66^Shc^ in the enhanced generation of ROS and in initiating apoptosis in hepatocytes after partial hepatectomy in aged mice, but not in the livers of young animals [29]. In this study, hepatocyte proliferation in both young and old cohorts was similar, but cell growth was impaired only in the old mice. Furthermore, ablation of p66^Shc^ diminished posthepatectomy oxidative stress and apoptosis in aged mice, suggesting that this age-associated protein may play a critical role in inhibiting the hepatic regenerative capacity in old animals.

Gielchinsky et al. recently reported an interesting observation that may have clinical ramifications for enhancing hepatic regeneration in the elderly, at least in elderly women [32]. These investigators reported that (a) the post-hepatectomy regenerative rate was restored in aged pregnant mice in comparison to their age-matched, nonpregnant cohorts and (b) this regeneration was achieved primarily by hepatocyte hypertrophy rather than by cell proliferation, the process responsible for normal liver regeneration. The clinical potential resides in the possible pharmacological activation of an important mediator of hepatocyte growth, the Akt/mTPRC1 pathway, and the subsequent switch from a cell proliferative to a cell growth response.

## 5. Age-Related Loss of Telomere Length

**Figure 3:**
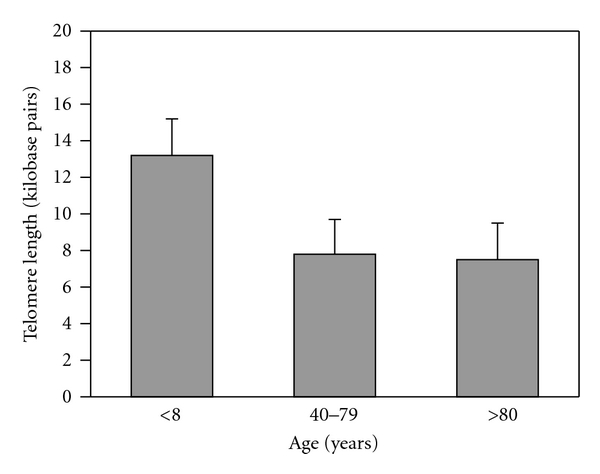
Effect of aging on telomere length in human hepatocytes. Both Takubo et al. and Aikata et al. (not shown) determined hepatocyte telomere length to be between 5–10 kbp in humans 80 years of age. Data derived from [26, 27].

Another hypothesis suggests that an age-related reduction in hepatocyte telomere length results in diminished cell mitosis and apoptosis and, thus, a decline in cell proliferation. For example, Takubo et al. demonstrated a marked age-associated loss in hepatocyte telomere length in humans, and these data were confirmed independently by Aikata et al. [26, 33] (Figure 3). Takubo et al. also reported that the rate at which telomere shortening occurred was markedly higher in hepatocytes in comparison to most other epithelial cell types with high turnover rates, for example, enterocytes and esophageal epithelium [26]. A recent review suggests that the yearly reduction rate in human hepatocyte's telomere length ranges between 55 and 120 base pairs [27]. 

Obvious changes in cell structure are not always reflected in concomitant functional alterations. Using a telomere restriction fragment deficient mouse model, Denchi et al. demonstrated that the loss of telomere integrity did not compromise liver regeneration following partial hepatectomy [35]. Although the hepatocytes enter S-phase, subsequent mitosis, anaphase, and telophase did not occur. This paper is of particular interest since it demonstrates that mouse hepatocytes subjected to the deletion of a telomere protection protein, TRF2, exhibit frequent telomere fusions, but no evidence of apoptosis or loss of hepatic function(s). Furthermore, post-hepatectomy regeneration was not compromised, but was accomplished via increased cell growth yielding polyploid cells, perhaps indicative of a switch from a proliferative to a cell growth pathway [32]. 

 Interestingly, our stereological analyses showed that mean hepatocyte volume in male F344 rats decreases between 20 and 30 months of age such that cells in the livers of the oldest animals were similar in volume to those in very young animals [5]. Our data also demonstrated that the relative number of binucleate hepatocytes, the nuclear numerical density, and the nucleocytoplasmic volume ratio were similar in the youngest and oldest rats. However, these studies were performed on resting hepatocytes, and the data do not preclude the possibility that small hepatocytes in senescent rats lack the capacity to undergo hypertrophy in response to mitogenic factors.

The recent observation that rejuvenating telomerase activity in a telomerase-deficient mouse model reversed certain well-documented age-related deficits may lend credence to further studies on liver regeneration in this model [36]. The caveat, however, is that aging in this particular telomerase-deficient mouse model may not correctly reflect normal human aging. A recent review by Hoare et al. provides a comprehensive discussion of the relationships between aging, hepatocyte telomere shortening, and hepatic injury or disease [37].

## 6. Effect of Aging on the Hepatocellular Response to Growth Factors

**Figure 4:**
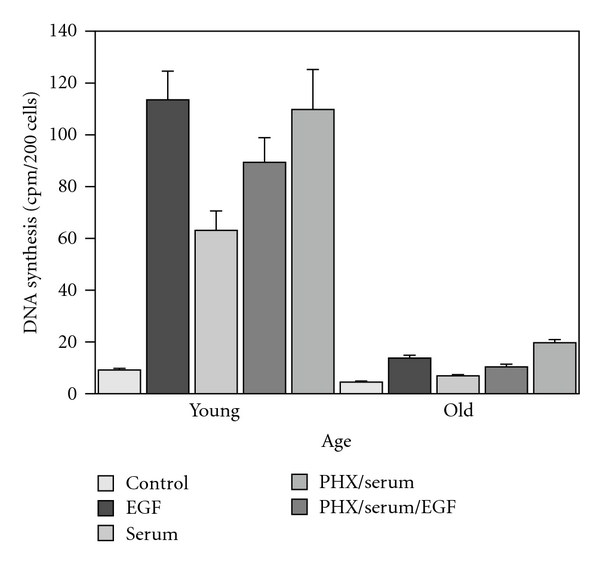
Effect of age on hepatocyte response to EGF and serum growth factors in young and old resting and posthepatectomy rats. Note that the posthepatectomy responses to both EGF (PHX) and EGF/serum were significantly greater in the young rats in comparison to their older cohorts. Data derived from [28].

Twenty years ago, Sawada demonstrated that the hepatocyte proliferative response to EGF was markedly greater in young rats in comparison to old animals and suggested that aging impaired the responsiveness of the cells in old rats to growth factors [28] (Figure 4). These studies provided impetus to the controversy concerning the impact of aging on the hepatocyte proliferative response to growth factors. For some years, researchers have suspected that aging impairs specific growth-regulating molecules and/or their receptors, which, in turn, compromises the regenerative response. Despite Sawada's observation that old hepatocytes did not respond to EGF stimulation as well as did liver cells from young animals, they also reported that there were no age-related losses in either the number of hepatocellular EGF receptors or in their binding affinity. However, Marti, in our laboratory, demonstrated a 60% age-related decline in EGF binding to hepatocyte plasma membranes in rats [38]. Interestingly, Ishigami et al. almost simultaneously reported the absence of any age-related change in hepatocyte EGF binding capacity, but did report a marked decline in EGF-induced DNA synthesis [39]. It should be noted that Ishigami et al. used primary hepatocyte cultures, whereas Marti et al. used hepatocyte plasma membranes isolated from intact livers. The preparation of primary hepatocyte cultures involves the use of collagenase and other enzymes that cleave hepatocyte surface proteins nonspecifically, for example, EGF receptors, resulting in cells from both young and old donors expressing equivalently diminished numbers of receptors. The isolation of hepatocellular plasma membranes does not employ enzymes, and the inherent number of receptors and, assumedly, their affinity for their ligand(s) remain intact during this procedure. Interestingly, we observed an 80% age-related decline in the amount of radiolabeled EGF associated with rat hepatocyte nuclei [40]. Furthermore, Ohtake et al. reported age-related losses of the hepatocyte high-affinity EGF receptor as well as in the level of receptor phosphorylation, a critical step in EGF activation [41].

**Figure 5:**
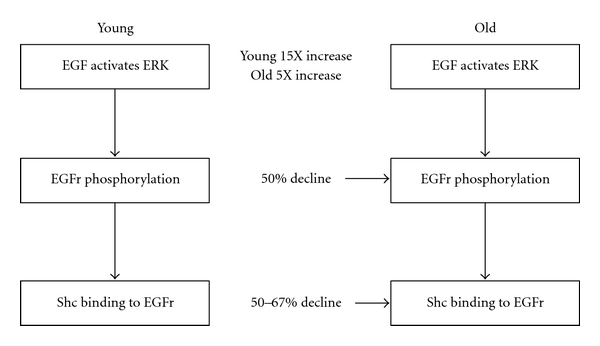
Effect of age on hepatic EGF receptor activation. Data derived from [30, 31].

**Figure 6:**
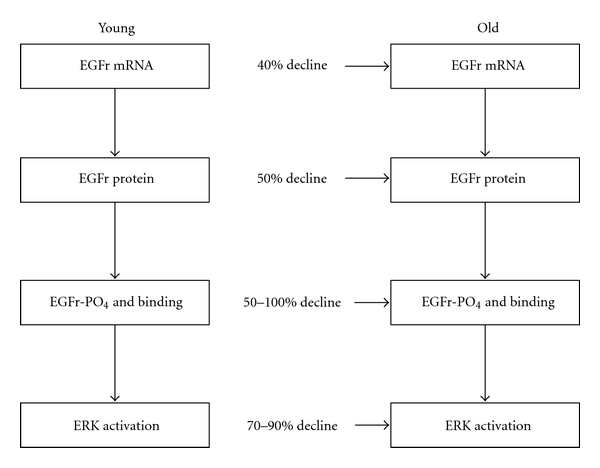
Effect of age on liver EGF activation, phosphorylation, and subsequent activation of the extracellular receptor kinase (ERK). Data derived from [34].

**Figure 7:**
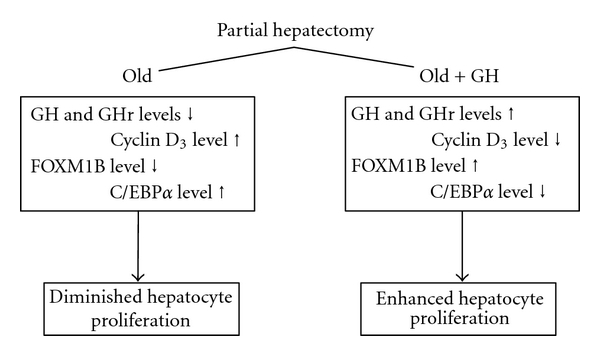
Effect of age on the efficacy of growth hormone induced activation of the pathway resulting in hepatocyte proliferation. Data derived from [34].

Several studies have reported diminished activation of a hepatocyte extracellular receptor kinase (ERK) in old rodents in comparison to young animals following partial hepatectomy [30, 31]. This decline leads to reduced EGF receptor phosphorylation and, subsequently, to decreased binding of the adapter protein, Shc, to the receptor, a critical event in the EGF-induced hepatocyte proliferation pathway (Figure 5). Subsequent studies by Kamat and others focused on the molecular pathways that regulate hepatocyte proliferation [34]. These investigators reported significant age-related declines in the expression of hepatocyte EGF receptor mRNA and protein, as well as in EGF receptor phosphorylation and the subsequent activation of ERK (Figures 6 and 7).

Growth hormone (GH) is another mitogenic factor that has been implicated in hepatic regeneration. Krupczak-Hollis et al. reported that GH treatment of old, partially hepatectomized rats enhances hepatocyte proliferation in comparison to similarly aged, nontreated cohorts [42]. Furthermore, the endogenous hepatocellular levels of GH and its receptor decline with age, whereas the level of cyclin D_3_, which activates C/EBP*α* (CCAAT/enhancer-binding proteins) phosphorylation, increases. This phosphorylation enhances C/EBP*α* complexing with (a) a retinoblastoma gene product, (b) a chromosomal remodeling protein (Brm), and (c) a histone deacetylase to yield an inhibitor of a transcription factor required for hepatocyte proliferation, the Forkhead Box gene, FOXM1B.

The importance of transcription factors in the liver regeneration process has been illustrated in a series of studies by Wang and associates delineating the critical role played by the FOXM1B gene in hepatocyte proliferation [43, 44]. Using a mouse model deficient in FOXM1B, these investigators showed that adenovirus transfection with FOXM1B restored the liver regenerative capacity in mature animals to a level that exceeded that measured in young adult mice. Nontransfected FOXM1B-deficient mice did not exhibit enhanced hepatocyte proliferation.

**Figure 8:**
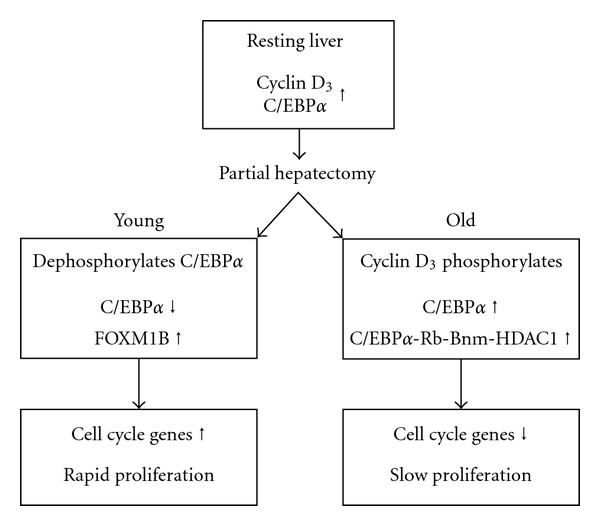
Proposed effect of aging on the molecular factors that regulates hepatocyte proliferation following partial hepatectomy.

In the resting liver, the hepatocellular levels of cyclin D_3_ and C/EBP*α* are high, thus inhibiting hepatocyte proliferation. In senescent animals, the cyclin D_3_ level remains high, activating the phosphorylation of C/EBP*α* and enhancing the formation of the larger proliferation inhibitory complex. However, following partial hepatectomy in young adult animals, the cyclin D_3_ level drops, as does the level of the inhibitor, C/EBP*α*, permitting the expression of essential transcription factors, for example, FOXM1B and cell cycle genes, and, ultimately, rapid hepatocyte proliferation (Figure 8).

Recently, Chen and colleagues identified a mechanism that regulates FOXM1B transcriptional activation and the liver regeneration process [45]. These researchers showed that the farnesoid X receptor (FXR), a transcription factor that regulates a variety of metabolic pathways, is critical for liver regeneration since FXR-deficient mice exhibit a diminished regenerative capacity. In addition, FOXM1B was identified as a direct FXR target gene, and diminished FXR binding to FOXM1B may contribute to decreased hepatocyte regeneration in the elderly.

## 7. Other Possible Causes of Diminished Regeneration

A series of studies by Le Couteur et al. reported marked age-related changes in the structure of the hepatic sinusoidal endothelium, including a loss of fenestrae and a thickening of the endothelial cells, a process referred to as pseudocapillarization [46–48]. Interestingly, a very recent paper by Furrer et al. has suggested that pseudocapillarization contributes to the age-related decline in the regenerative response in a posthepatectomized murine model [49]. These investigators demonstrated enhanced liver regeneration in old mice following treatment with a serotonin receptor agonist, and this response correlated with an increase in the number of endothelial cell fenestrae. Their data suggest that the serotonin receptor agonist enhances systemic vascular endothelial growth factor (VEGF) availability, which, in turn, regulates endothelial cell fenestrae diameters, thus improving hepatic perfusion and restoring the hepatic regenerative capacity. However, evidence for or against age-related declines in serotonin and/or VEGF receptors will require definitive ligand-binding studies. 

In summary, there are several conclusive statements and a few evidence-based speculations concerning the effect of age on the process of liver regeneration that warrant consideration, including the following.

- Liver regeneration is compromised in old animals and in elderly humans.
- The rate of liver regeneration, rather than the regenerative capacity, is diminished in the elderly.
- The induction of hepatocyte proliferation factors and the expression of cell cycle genes is inhibited in the elderly.
- The repression of cell proliferation and cell cycle gene inhibitors is compromised in the elderly
- The relative efficacies of normal, cell-cycle-induced hepatocyte proliferation versus an independent pathway involving hepatocyte hypertrophy in maintaining liver functions requires additional study.
- The roles of VEGF, serotonin, and liver sinusoidal pseudocapillarization require further investigation.

Most evidence supports the concept that the age of the liver donor or recipient exerts only a modest impact on post-transplantation patient's survival. These studies suggest that a pretransplantation regimen of growth factors in potential elderly liver recipients merits further consideration.
